# PeachMD: a multi-omics database for peach

**DOI:** 10.1186/s43897-025-00157-z

**Published:** 2025-07-04

**Authors:** Ang Li, Shihang Sun, Hongmei Wang, Akhi Badrunnesa, Junren Meng, Xiongwei Li, Yuan Gao, Liang Niu, Lei Pan, Wenyi Duan, Guochao Cui, Zhiqiang Wang, Wenfang Zeng

**Affiliations:** 1 Zhengzhou Fruit Research Institute, Chinese Academy of Agricultural Sciences, Zhengzhou, 450009 PR China; 2 Zhongyuan Research Center, Chinese Academy of Agricultural Sciences, Xinxiang, 453500 China; 3 Forest & Fruit Tree Institute, Shanghai Academy of Agricultural Sciences, Shanghai, 201403 China

Peach *(Prunus persica* L. Batsch) is the most important temperate fruit crop globally. As a model species for the genus *Prunus* and other perennial fruit trees in the Rosaceae family, it has gattracted considerable attention in both biological and agricultural research. So far, 12 peach genomes have been sequenced and annotated, providing accurate data support for the genetic basis of peaches. Recent advancements in sequencing technology have enabled the comprehensive characterization of multi-omics data, such as genome sequencing, whole genome resequencing, transcriptomes, and whole genome bisulfate sequencing (WGBS), for the peach genome. However, this wealth of data is scattered across various databases, complicating rapid and efficient access and utilization. Additionally, analyzing such large-scale datasets requires considerable time and effort, posing a significant challenge for research teams engaged in wet lab experiments or those with limited computational resources. To address this, we have developed the first comprehensive Peach Multi-omics Database (PeachMD, http://www.peachmd.com). PeachMD offers extensive multi-omics data and user-friendly tools for their utilization. Thus, it is anticipated to be an invaluable resource for advancing molecular breeding and functional genomics research in peach, providing robust support for studies in related fields.

PeachMD is the most comprehensive peach database available globally, providing researchers with a rich and diverse array of data resources. It is constructed on the MySQL and Flask framework in python, utilizing an efficient Nginx architecture to ensure fast data access and stability. The current version of PeachMD contains 12 reference genomes, 329 transcriptome data, 101 WGBS data, and 1313 whole genome resequencing datasets from published literature and our laboratory (Fig. S1). To eliminate errors caused by different analytical methods, a unified process was employed to reanalyze these datasets. The specific methods have been added to the supplementary information. To facilitate data utilization, 852 germplasms have been cataloged, along with visualized Genome-wide association studies (GWAS) results for exploring genes associated with important agronomic traits in peaches. In the reverse genetics approach, transcriptome and epigenetic data from different samples are used to explore the molecular mechanisms behind phenotypes. In addition, several useful tools are also provided, including Primer3, JBrowse, BLAST, TF/TR, Gene LiftOver, KEGG/GO Enrichment, Genome Synteny, and CRISPR Design.

In the Genomes module, we have collected all 12 published genomes from various sources, including 4 improved accessions, 4 landraces and 4 wild types (Cao et al. 2022; Cao et al. 2021; Guan et al. 2021; Lian et al. 2022; Verde et al. 2017; Wang et al. 2023; Zhou et al. 2021; Zhou et al. 2023). The Gene Index and Gene Index Multi functions enable users to retrieve genome annotation and functional annotation data for single genes or multiple genes from a variety of databases. They can also download promoter sequences (2 kb upstream of TSS), CDS, and protein sequences for all reference genomes. Furthermore, gene structure information is available for visualization using JBrowse (Fig. 1A). The Genome Synteny function enables users to easily select any two reference genomes to visualize their genomic collinear blocks and collinear genes within the database, which is essential for analyzing gene gain and loss. Transposable Elements (TEs), as widespread components of plant genomes, may drive genomic variation and significantly contribute to the rapid adaptation and domestication of plants. Therefore, we have developed the Transposable element (TE) section, which provides TE classification, sequences, and position information (Fig. 1B).

**Fig. 1:**
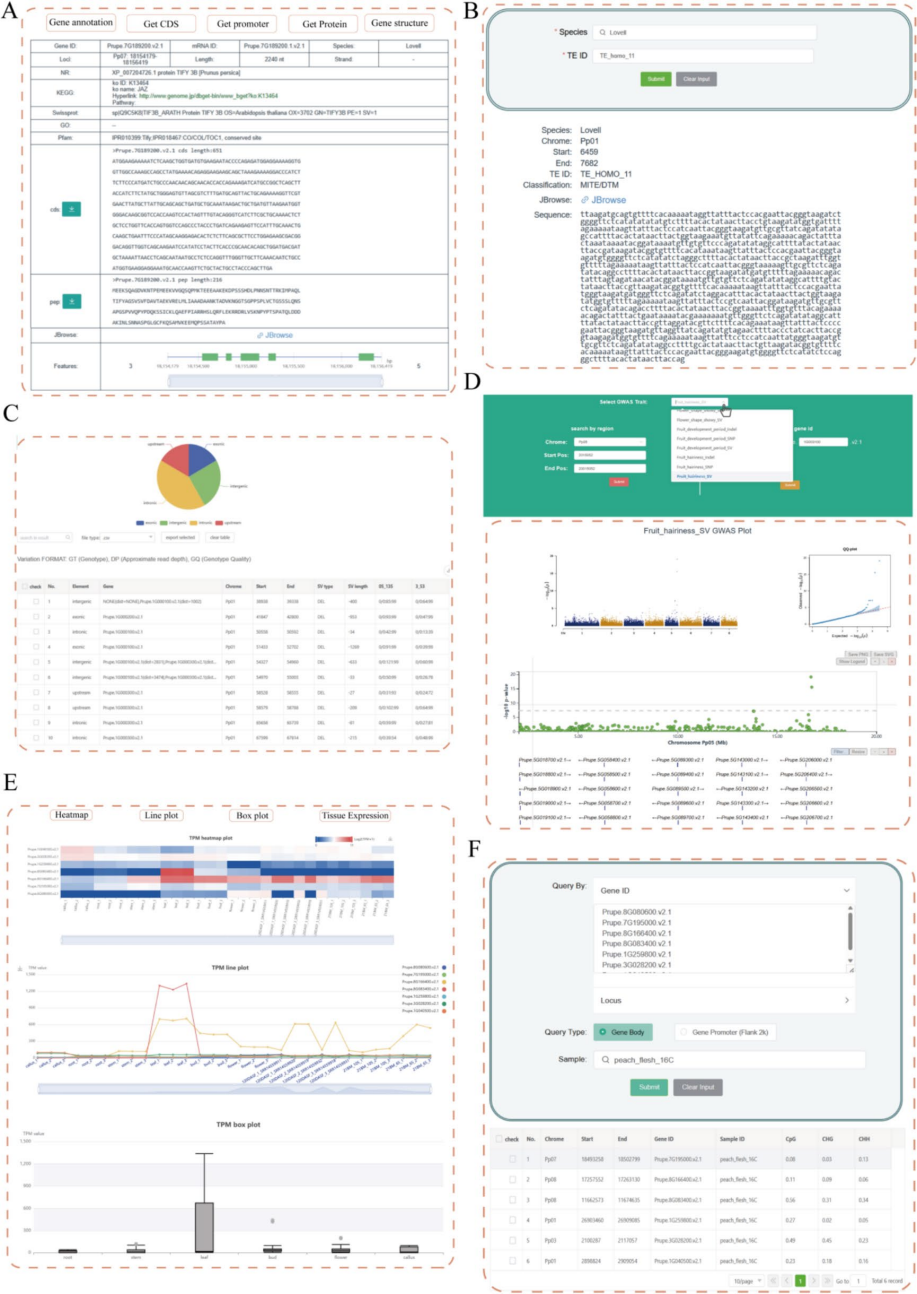
Screenshots of the PeachMD database. **A** Gene index module. **B** Transposable element (TE) module (**C**) Variation module. **D** GWAS module. **E** Transcriptome module. **F** DNA methylation module

At the population level, we have collected 852 varieties from global sources in the Germplasm function, and this module contains 13 germplasm phenotype records, including fruit weight, soluble solids content (%), chilling requirement (h), flesh adhesion, fruit hairiness, fruit shape, flesh color, flower shape (double/single; showy/ non-showy), pollen fertility, anther color, fruit development period and development period. This collection can be visualized on a world map with details on the sources provided. It also provides a search tool for users to quickly query the traits of these germplasm. In addition, the module also mainly includes whole genome resequencing data of 1313 peach accessions from previously published studies. A query system enables rapid identification of SNPs, Indels, and SVs by gene ID or genomic positions in different samples (Fig. 1C). Furthermore, GWAS information on important agronomic traits of peach has been collected to identify candidate genes responsible for various traits (Guo et al. 2020). This page presents SNPs, Indels and SVs with statistically significant associations with 26 agronomic traits in the form of Manhattan plots and annotation tables (Fig. 1D). The Evolution module provides selection signals, including nucleotide diversity (*Pi*), fixation index (*FST*), and XPCLR, for detecting the genomic regions or genes under selection.

The relationship between gene expression and function is closely interconnected, and studying gene expression helps us understand gene functions and discover the underlying regulatory networks. We collected a total of 329 transcriptomic libraries, with 57 libraries generated by our lab and the remaining data obtained from NCBI (https://www.ncbi. nlm.nih.gov/). The Transcriptome page provides a user-friendly interface for searching by FPKM, TPM, and read count, along with various visualization options including tables, heatmaps, and line plots (Fig. 1E). This flexible search capability enables researchers to quickly find expression data related to specific genes or conditions. Additionally, box plots are utilized to illustrate the expression levels of selected transcripts across different tissues. This method effectively highlights the expression differences among various tissues, revealing the functional characteristics of genes within specific biological contexts, thereby providing important support for subsequent functional validation and gene research.

The Tools module encompasses a total of 10 tools. The TF/TR section presents comprehensive data on 70 transcription factor families and 24 transcriptional regulators across all reference genomes. The DNA methylome section enables users to assess DNA methylation levels in CG, CHG, and CHH contexts within gene and promoter regions (Fig. 1F). The Enrichment, BLAST, Primer3, Gene LiftOver, and CRISPR Prime tools permit users to conduct online analyses with their datasets.

Overall, PeachMD is a multifaceted interactive platform designed specifically for researchers focused on peaches. PeachMD integrates cutting-edge genome sequences and omics data, providing essential resources and tools for the swift identification of candidate genes, validation of gene functions, elucidation of how genetic variations influence gene expression and traits, and guidance on optimal breeding strategies. The streamlined user interface of PeachMD enhances usability, and the detailed user manual provided allows users to conveniently and efficiently access data, thus supporting their research activities. In addition, we have provided a case study to demonstrate a method that integrates multi-omics data (Fig.S2). PeachMD is an ongoing, dynamic project that will receive regular updates with the new reference genome assemblies and additional omics datasets. We also welcome valuable feedback from our peers to help us continuously enhance PeachMD.

## Supplementary Information

Supplementary Material 1: Fig. S1. Basic schema and data source of PeachMD.

## Data Availability

The data will be available from the corresponding author upon reasonable request.
