# Supplementary material for: PeachMD: a multi-omics database for peach
Source: Mol Hortic. 2025 Jul 4;5:37. doi: 10.1186/s43897-025-00157-z (PMC12232059; doi:10.1186/s43897-025-00157-z)
Supplement: Supplementary file 1 — Supplementary Material 1: Fig. S1. Basic schema and data source of PeachMD. [file 43897_2025_157_MOESM1_ESM.docx]

**Supplemental data**

**Supplemental materials and methods**

**Genome annotation analyses**

Peach genome sequences, gff files, and protein sequences were downloaded from public databases such as NCBI (https://www.ncbi.nlm.nih.gov/), CNCB (https://www.cncb.ac.cn/) and GDR (https://www.rosaceae.org/). The functions of genes were annotated using four protein databases, namely the EggNOG-mapper (http://eggnog-mapper.embl.de/), KEGG (https://www.genome.jp/kegg/), KOBAS3.0 (http://kobas.cbi.pku.edu.cn/), and Pfam databases (http://pfam-legacy.xfam.org/). EDTA v2.2.1 (Ou et al., 2019) was used to perform a preliminary identification of transposons in each genome, and DeepTE (Yan et al., 2020) software was used to classify unknown types of transposons based on the above identification results, and then further identification was performed using EDTA software to ensure the reliability of the results. In addition, TF/TR were further identified and annotated by iTAK v2.0.2 (Zheng et al., 2016) software.

**Synteny analysis**

The synteny analysis was carried out using JCVI v1.4.21 (Tang et al., 2024). For the collinearity analysis within the *Rosaceae* family, we selected the Fragaria_vesca (https://www.rosaceae.org/species/fragaria_vesca/genome_v4.0.a1), Malus _domestica (https://www.rosaceae.org/species/malus/malus_x_domestica/genome_GDDH13_v1.1), Prunus_avium (https://www.rosaceae.org/Analysis/9262820), Prunus_dulcis (https://www.rosaceae.org/Analysis/20220996), Prunus_salicina (https://www.rosaceae.org/Analysis/9450778), Rosa_wichuraiana (https://www.rosaceae.org/Analysis/13087667), and Rubus_occidentalis (https://www.rosaceae.org/analysis/268) genomes.

**Transcriptome sequencing data analysis**

Fastp v0.23.4 was used to filter the raw reads and then generate clean data (Chen et al. 2018). These clean reads were mapped to the ‘lovell’ genome using HISAT2 v2.2.1 (Kim et al. 2019). Gene expression was calculated by Subread v2.0.3 (Liao et al., 2013).

**Resequencing data analysis**

Raw Illumina reads were processed to remove adapter and low-quality sequences by Fastp v0.23.4 (Chen et al. 2018). The paired-end reads were mapped to the reference peach ‘Lovell’ genome using BWA v0.7.18 (Li et al,. 2010) with the following parameters: ‘bwa mem -M -R.’, and sorting the mapped reads according to genomic coordinates using Samtools v1.21 (Li et al., 2009). Reads from different Illumina lanes were merged using ‘samtools merge.’ Subsequently, duplicate reads from PCR were removed with Picard v2.25.5. Finally, the HaplotypeCaller mode of GATK v4.4.0.0 (McKenna et al., 2010) software is used to identify SNP/INDEL mutation sites with default parameters, and finally the original mutation data set is obtained. Due to differences in the depth of resequencing among populations, we used the --minQ parameter to filter SNPs (--minQ 180) and INDELs (--minQ 50) rseparately, and annotated all the mutation sites by ANNOVAR v2013-06-21 (Wang et al., 2010). At the same time, structural variations were identified using the default parameters of the LUMPY v0.3.1 (Layer et al., 2014). The SV results were then genotyped in the population using SVTyper v0.0.4 (Chiang et al., 2014).

**Genetic diversity and selection signature analysis**

Only SNPs with a minor allele frequency greater than 0.05 and a missing rate greater than 0.8 were retained; genetic differentiation (*Fst*) for each pair of citrus populations, allele frequency, and nucleotide diversity (Π) in each peach population were calculated by VCFtools v0.1.16 (Lunter et al., 2011) using a window size of 10 kb and a step size of 1 kb. XP-CLR is a method that uses allele frequency differentiation at linked loci between two populations to detect selective sweeps. Each chromosome was analyzed using the program XPCLR v1.0 (Chen et al., 2010), and the average XP-CLR scores were calculated for each 10 kb sliding window with a step size of 1 kb.

**Germplasm data**

Data on 13 germplasm phenotype from 852 germplasm collections were obtained from the book *peach genetic resource in China* (Wang et al., 2012)*.*

**GWAS for 26 agronomic traits**

To improve the accuracy of the GWAS results, we filtered the SV, Indel, and SNP datasets by removing those with minor allele frequency (MAF) < 0.01. We use the mixed linear model program in tassel v.5.2.13 (Bradbury et al., 2007). In this page reports SNPs, Indels and SVs with statistically significant associations with 26 agronomic traits in the form of Manhattan and QQ plots.

**Whole-genome bisulfite sequencing data analysis**

Fastp v0.23.4 was used to filter raw data reads to obtain clean reads. Bismark v0.22.3 (Krueger and Andrews 2011) with default parameters was used to align the bisulfite treated clean data to the ‘lovell’ genome. DNA methylation level was calculated by BatMeth2 v1.1 (Zhou et al., 2019).

**Integration of other biological analysis tools**

We offer several practical bioinformatics tools. Applying the method proposed in CRISPR-P (Liu et al., 2017) to our websites generates the CRISPR primer tool, which finds all the target sites with protospacer adjacent motif sequences in the search region. In addition, the more user-friendly and responsive JBrowse2 (Diesh et al., 2023) technology was deployed in our website for the presentation of multi-omics data. Simple and useful tools Blast (Camacho et al., 2009), Primer (https://primer3.ut.ee/), Pfam (http://pfam-legacy.xfam.org/) and GO/KEGG Enrichment (Yu et al., 2012) are also deployed in PeachMD.

**Database construction**

PeachMD is constructed on the MySQL (https://www.mysql.com/) and Flask framework in python (https://flask.palletsprojects.com/), utilizing Nginx architecture (https://nginx.org/en/) and is deployed on CentOS 7.6 system provided by Tencent Cloud (https://cloud.tencent.com/). PeachMD has been extensively tested on major web browsers such as IE/Edge, Firefox, Google Chrome and Safari. In addition, our website was designed with global users in mind, ensuring accessibility for both Chinese and overseas users. These tests demonstrate the stable performance of the website and ensure international availability.

**Case study: Characterization of a NAC transcription factor related to peach fruit ripening**

PeachMD aims to establish a comprehensive one-stop multi-omics data analysis platform for peaches. This platform is designed to integrate various types of omics data, including genomics, transcriptomics, epigenomics, and more, providing researchers with a powerful tool to explore the complex biological processes underlying peach growth, development, and response to environmental stresses. To demonstrate the outstanding capabilities of PeachMD and the practical application of related tools, we conducted an in-depth case study focusing on the interplay between gene regulation and epigenetic modifications during peach fruit maturation.

Fruit ripening is a multifaceted biological process encompassing hormone synthesis, pigment accumulation, textural transformation and softening, as well as the generation of flavor-related compounds such as soluble sugars, organic acids, and aromatic volatiles (Tang et al., 2020). The plant-specific NAC (NAM/ATAF1/2/CUC2) transcription factor family is crucial for regulating fruit ripening in both climacteric and nonclimacteric fruits (Olsen et al., 2005). In previous studies, it was found that PpNAC1 is closely related to peach ripening, participating in ethylene synthesis, pectin metabolism, sugar transport, and volatile organic compound synthesis (Cao et al., 2023).

Utilizing PeachMD, we first used the TF/TR tool to search for all 114 NAC members in the peach genome (Fig.S2A). Blast results show that PpNAC1 has 56% similarity at the amino acid sequence to tomato NAC-NOR (Fig. S2B), and using the gene index tool, we queried the annotations, sequence information, and gene structure of PpNAC1 (Fig. S2C). Next, we further analyzed the expression pattern of *PpNAC1.* It is worth noting that PpNAC1 exhibits a similar expression pattern during fruit ripening in different peach varieties, with transcription levels increasing as the fruit ripens (Fig. S2D).

DNA methylation is considered a heritable epigenetic modification that silences TEs, while also playing a role in maintaining genome stability, gene imprinting, and regulating gene expression (Bartels et al., 2018). Numerous studies have shown that DNA methylation is closely related to fruit ripening, and the methylation level of the promoter region is negatively correlated with gene expression (Cao et al., 2023). Conducting integrated analysis of genome, TEs, and methylation through the genome browser in PeachMD revealed intriguing details (Fig. S2E). As the peach fruit matures, the methylation level of the *PpNAC1* promoter region decreases, and this region overlaps with TE (TE_00002365). Further use of TE tools can obtain classification and sequence information of TEs (Fig. S2F). Thus, these new findings provide rationales for designing experiments to further validate the findings.

References

Abyzov A., Urban A.E., Snyder M., Gerstein M. CNVnator: An approach to discover, genotype, and characterize typical and atypical CNVs from family and population genome sequencing. Genome Res. 2011;21(6):974-84. <https://doi.org/10.1101/gr.114876.110>

Bradbury P.J., Zhang Z., Kroon D.E., Casstevens T.M., Ramdoss Y., Buckler E.S. TASSEL: software for association mapping of complex traits in diverse samples. Bioinformatics. 2007;23(19):2633-2635. <https://doi.org/10.1093/bioinformatics/btm308>

Bartels A., Han Q., Nair P., Stacey L., Gaynier H., Mosley M., et al. Dynamic DNA Methylation in plant growth and development. IJMS. 2018;19(7):2144. https://doi.org/10.3390/ijms19072144.

Camacho C., Coulouris G., Avagyan V., Ma N., Papadopoulos J., Bealer K., et al. BLAST+: architecture and applications. BMC Bioinform. 2009;10:421. <https://doi.org/10.1186/1471-2105-10-421.>

Cao X., Li X., Su Y., Zhang C., Wei C., Chen K., et al. Transcription factor PpNAC1 and DNA demethylase PpDML1 synergistically regulate peach fruit ripening. Plant Physiol. 2023;194(4):2049-2068. https://doi.org/10.1093/plphys/kiad627

Chen H., Patterson N., Reich D. Population differentiation as a test for selective sweeps. Genome Res. 2010;20(3):393–402. https://doi.org/10.1101/gr.100545.109

Chen, S., Zhou, Y., Chen, Y., Gu, J. fastp: an ultra-fast all-in-one FASTQ preprocessor. Bioinformatics. 2018;34(17):i884-i890. <https://doi.org/10.1093/bioinformatics/bty560>

Chiang C., Layer R.M., Faust G.G., Lindberg M.R., Rose D.B., Garrison E.P., et al. SpeedSeq: Ultra-fast personal genome analysis and interpretation. Nat Methods. 2015;12:966-8. <https://doi.org/10.1101/012179>

Danecek P., Auton A., Abecasis G., Albers C.A., Banks E., DePristo M.A., et al. The variant call format and VCFtools. Bioinformatics. 2011;27(15):2156-2158. <https://doi.org/10.1093/bioinformatics/btr330>

Diesh C., Stevens G.J., Xie P., De Jesus Martinez T., Hershberg E.A., Leung A., et al. JBrowse 2: a modular genome browser with views of synteny and structural variation. Genome Biol. 2023;24(1): 1-21. https://10.1186/s13059-023-02914-z

Kim, D., Langmead, B., Salzberg, S. L. HISAT: a fast spliced aligner with low memory requirements. Nature Methods. 2015;12(4):357-360. <https://doi.org/10.1038/nmeth.3317>

Krueger, F., & Andrews, S. R. Bismark: a flexible aligner and methylation caller for Bisulfite-Seq applications. Bioinformatics. 2011;27(11):1571-1572. <https://doi.org/10.1093/bioinformatics/btr167>

Larson D.E., Abel H.J., Chiang C., Badve A., Das I., Eldred J.M., et al. svtools: population-scale analysis of structural variation. Bioinformatics. 2019;35(22):4782-4787. <https://doi.org/10.1093/bioinformatics/btz492>

Layer, R. M., Chiang, C., Quinlan, A. R., Hall, I. M. LUMPY: a probabilistic framework for structural variant discovery. Genome Biol. 2014;15(6):R84. <https://doi.org/10.1186/gb-2014-15-6-r84>

Li, H., Durbin, R. Fast and accurate long-read alignment with Burrows–Wheeler transform. Bioinformatics. 2010;26(5):589-595. <https://doi.org/10.1093/bioinformatics/btp698>

Li H., Handsaker B., Wysoker A., Fennell T., Ruan J., Homer N., et al. The Sequence Alignment/Map format and SAMtools. Bioinformatics. 2009;25(16):2078-2079. <https://doi.org/10.1093/bioinformatics/btp352>

Liao, Y., Smyth, G. K., Shi, W. The Subread aligner: fast, accurate and scalable read mapping by seed-and-vote. Nucl Acids Res. 2013;41(10):e108-e108. <https://doi.org/10.1093/nar/gkt214>

Liu H., Ding Y., Zhou Y., Jin W., Xie K., Chen L.-L. CRISPR-P 2.0: An Improved CRISPR-Cas9 Tool for Genome Editing in Plants. Mol Plant. 2017;10(3):530-532. https://doi.org/j.molp.2017.01.003

McKenna A., Hanna M., Banks E., Sivachenko A., Cibulskis K., Kernytsky A., et al. The Genome Analysis Toolkit: A MapReduce framework for analyzing next-generation DNA sequencing data. Genome Res. 2010;20(9): 1297-1303. <https://doi.org/10.1101/gr.107524.110>

Olsen A.N., Ernst H.A., Leggio L.L., Skriver K. NAC transcription factors: structurally distinct, functionally diverse. Trends Plant Sci. 2005;10(2), 79-87. https://doi.org/10.1016/j.tplants.2004.12.010

Ou S., Su W., Liao Y., Chougule K., Agda J.R.A., Hellinga A.J., et al. Benchmarking transposable element annotation methods for creation of a streamlined, comprehensive pipeline. Genome Biol. 2019;20(1). <https://doi.org/10.1186/s13059-019-1905-y>

Tang D., Gallusci P., Lang Z. Fruit development and epigenetic modifications. New Phytol. 2020;228(3):839-44. https://doi.org/10.1111/nph.16724

Tang H., Krishnakumar V., Zeng X., Xu Z., Taranto A., Lomas J.S., et al. JCVI: A versatile toolkit for comparative genomics analysis. Imeta. 2024;3(4):e211. https://doi.org/10.1002/imt2.211

Wang L.，Zhu G.，Fang Wei．Peach genetic resource in China．Beijing：China Agricultural Press;2012．

Wang, K., Li, M., Hakonarson, H. ANNOVAR: functional annotation of genetic variants from high-throughput sequencing data. Nucl Acids Res. 2010;38(16):e164-e164. <https://doi.org/10.1093/nar/gkq603>

Yan, H., Bombarely, A., Li, S. DeepTE: a computational method for de novo classification of transposons with convolutional neural network. Bioinformatics. 2020;36(15):4269-4275. <https://doi.org/10.1101/2020.01.27.921874>

Yu G., Wang L.-G., Han Y., He Q.-Y. clusterProfiler: an R Package for Comparing Biological Themes Among Gene Clusters. OMICS. 2012;16(5):284-7. https://doi.org/10.1089/omi.2011.0118

Zheng Y., Jiao C., Sun H., Rosli H.G., Pombo M.A., Zhang P., et al. iTAK: A Program for Genome-wide Prediction and Classification of Plant Transcription Factors, Transcriptional Regulators, and Protein Kinases. Mol. Plant. 2016;9(12):1667-1670. <https://doi.org/10.1016/j.molp.2016.09.014>

Zhou Q., Lim J.-Q., Sung W.-K., Li G. An integrated package for bisulfite DNA methylation data analysis with Indel-sensitive mapping. BMC Bioinform. 2019;20(1). <https://doi.org/10.1186/s12859-018-2593-4>

**Supplemental Figures**


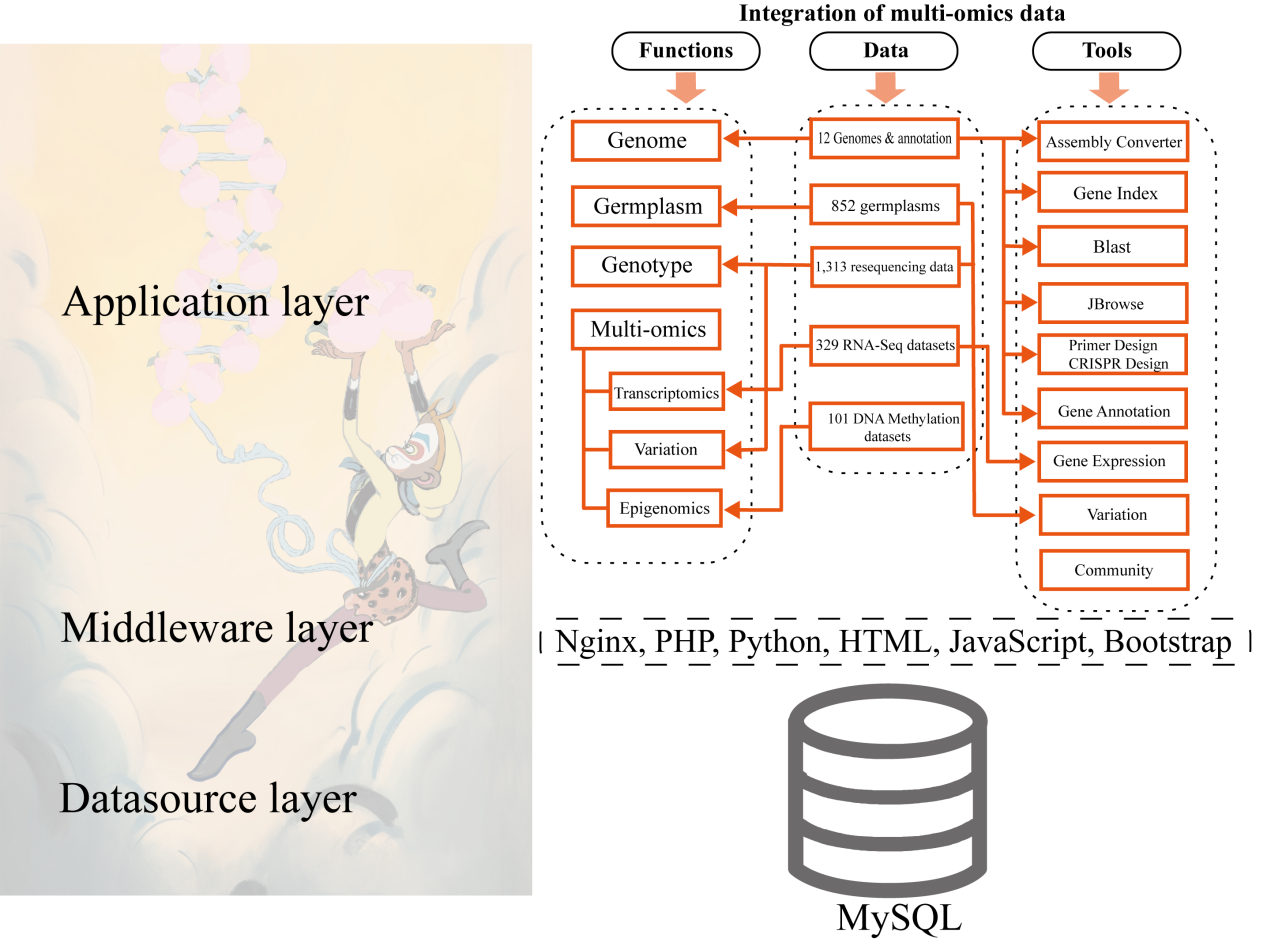


Fig.S1 Basic schema and data source of PeachMD.


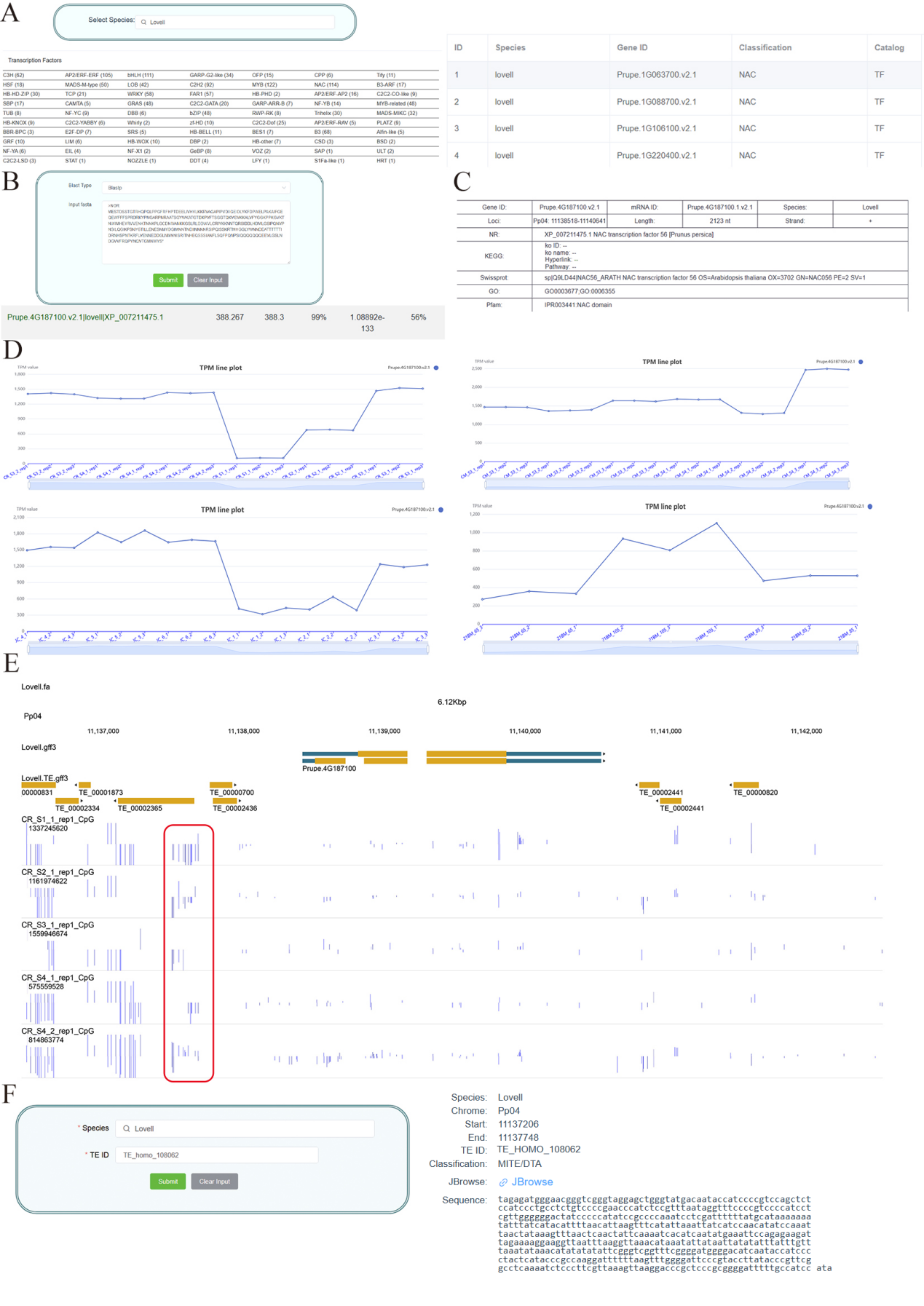


Fig.S2. An illustrative analysis outcome of PpNAC1 within PeachMD. (A) The members of NAC family in the peach genome. (B) Blast searches for homologous genes of SlNOR. (C) The basic sequence information of PpNAC1. (D) Expression profiles of PpNAC1 across different RNA-seq experiments during peach fruit development and ripening. (E) A snapshot of the genome browser for PpNAC1 illustrates the integrated analysis of the genome, methylation, and TEs. (F) A sample output for TE analysis.
